# Serum IgA Fc effector functions in infectious disease and cancer

**DOI:** 10.1111/imcb.12306

**Published:** 2020-01-19

**Authors:** Samantha K Davis, Kevin J Selva, Stephen J Kent, Amy W Chung

**Affiliations:** ^1^ Department of Microbiology and Immunology The Peter Doherty Institute for Infection and Immunity The University of Melbourne Parkville VIC Australia; ^2^ Melbourne Sexual Health Centre Infectious Diseases Department Alfred Health Central Clinical School Monash University Melbourne VIC Australia; ^3^ ARC Centre of Excellence in Convergent Bio‐Nano Science and Technology University of Melbourne VIC Australia

**Keywords:** Bacteria, CD89, Fc receptor, IgA, infectious disease, ITAM, ITAMi, serum, virus

## Abstract

Immunoglobulin (Ig) A is the most abundant antibody isotype present at mucosal surfaces and the second most abundant in human serum. In addition to preventing pathogen entry at mucosal surfaces, IgA can control and eradicate bacterial and viral infections through a variety of antibody‐mediated innate effector cell mechanisms. The role of mucosal IgA in infection (e.g. neutralization) and in inflammatory homeostasis (e.g. allergy and autoimmunity) has been extensively investigated; by contrast, serum IgA is comparatively understudied. IgA binding to fragment crystallizable alpha receptor plays a dual role in the activation and inhibition of innate effector cell functions. Mounting evidence suggests that serum IgA induces potent effector functions against various bacterial and some viral infections including *Neisseria meningitidis* and rotavirus. Furthermore, in the era of immunotherapy, serum IgA provides an interesting alternative to classical IgG monoclonal antibodies to treat cancer and infectious pathogens. Here we discuss the role of serum IgA in infectious diseases with reference to bacterial and viral infections and the potential for IgA as a monoclonal antibody therapy.

## Graphical abstract

The role of mucosal immunoglobulin A (IgA) in infection (e.g. neutralization) has been extensively investigated; by contrast, serum IgA is poorly understood. Crosslinking of serum IgA with fragment crystallizable alpha receptor I (FcαRI) can activate immune cells (e.g. phagocytosis, antibody‐dependent cellular cytotoxicity, reactive oxygen species) to clear select bacteria, viruses and tumors, whereas monomeric serum IgA can inhibit these functions hindering an effective immune response. Here we discuss serum IgA Fc effector functions in infectious disease and tumor clearance, potential applications in immunotherapy and limitations of current research.

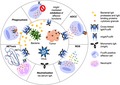

## Introduction

Immunoglobulins are involved in the control and clearance of infectious diseases including viral (e.g. HIV), bacterial (e.g. *Mycobacterium tuberculosis, N. meningitidis*) and parasitic pathogens (e.g. *Plasmodium* spp., *Leishmania* spp.) via various different mechanisms such as neutralization, and fragment crystallizable (Fc) effector functions including antibody‐dependent cellular cytotoxicity (ADCC), phagocytosis and complement activation.^1^ Immunoglobulin (Ig) G has been extensively studied and this is highlighted by the dozens of IgG monoclonal antibodies (mAbs) approved for therapeutic use by the US Food and Drug Administration.^2^ Recently, there has been a growing appreciation for other antibody isotypes including IgA as mAb therapeutics for cancer treatment and some viral and bacterial pathogens.^3^, ^4^, ^5^ IgA can neutralize invading pathogens and induce a range of Fc effector functions to control and clear various bacterial (e.g. *N. meningitidis* and *Streptococcus pneumoniae*) and viral infections (e.g. rotavirus and HIV).^4^, ^6^, ^7^, ^8^, ^9^, ^10^ Furthermore, IgA maintains homeostasis of inflammation at mucosal surfaces and in the blood and tissues.^11^ Mucosal IgA is important for first‐line defense from invading pathogens at mucosal surfaces. However, the role of serum IgA and associated Fc functions in infectious disease is incomplete and understudied. Here we will discuss serum IgA Fc effector functions in the context of control and elimination of invasive pathogens.

## IgA Structure

**Figure 1:**
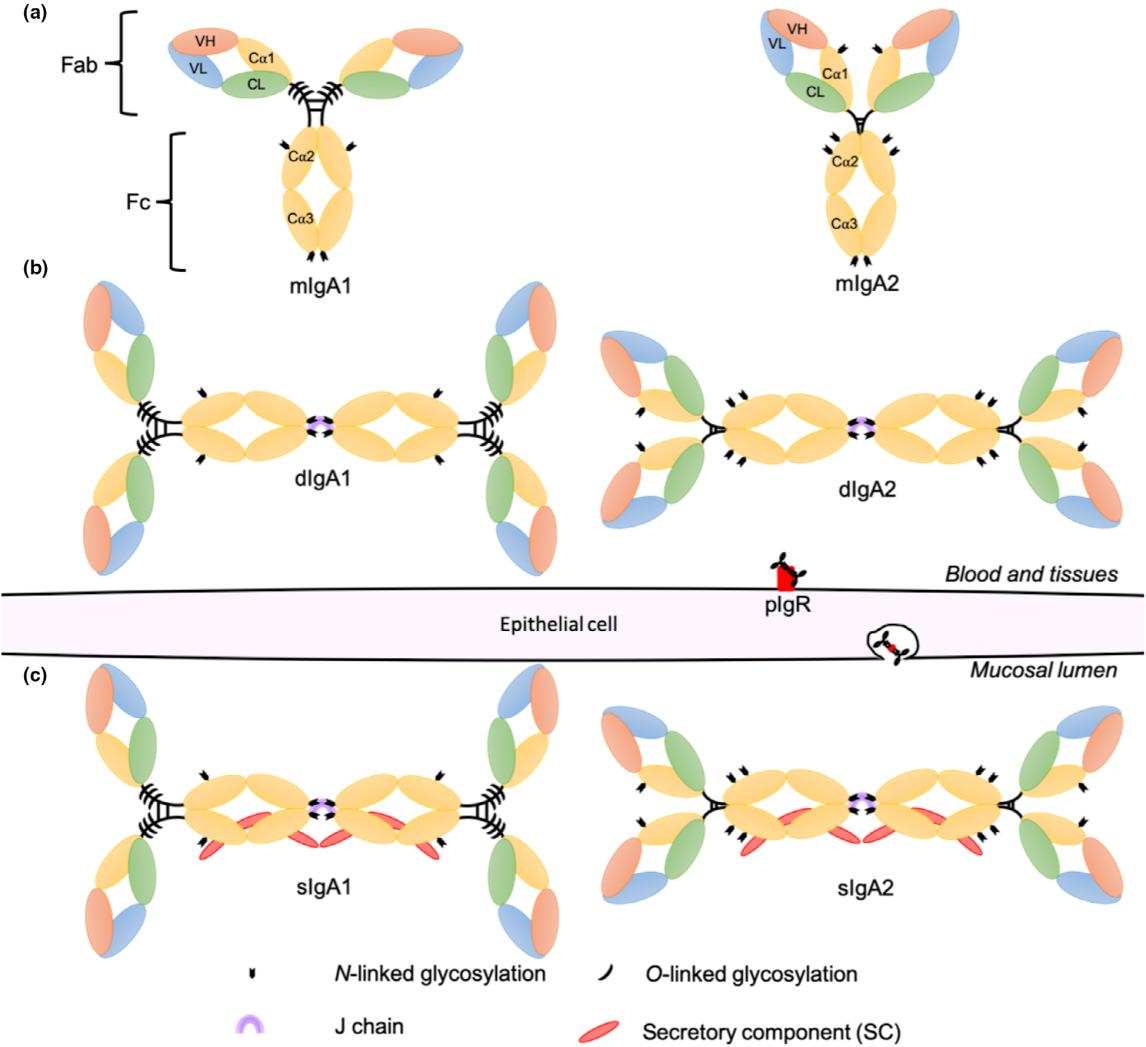
Schematic diagram of immunoglobulin A (IgA) subclasses IgA1 and IgA2, glycosylation patterns and their respective heterogenous molecular forms. In blood and tissue compartments **(a)** monomeric IgA (mIgA) and to a lesser extent **(b)** dimeric IgA (dIgA) [two IgA monomer Fc portions connected via a joining (J) chain] are present. dIgA is secreted through epithelial cells via the polymeric immunoglobulin receptor (pIgR) into the mucosal lumen with secretory component (SC) to form **(c)** secretory IgA (sIgA).

The five human antibody isotypes (IgG, IgA, IgE, IgD and IgM) mediate an array of functional activities. IgA is the most abundant antibody at mucosal surfaces, and the second most abundant in serum (~15%; 2–3 mg mL^−1^) behind IgG (80%; ~10–20 mg mL^−1^).^12^ More IgA is synthesized per day than all other antibody isotypes combined (66 mg^−1^ mL^−1^ day^−1^);^12^ however, rapid catabolism of serum IgA results in a relatively short half‐life (4–6 days).^13^ IgA consists of the typical monomeric antibody structure (see the “Future Directions and Conclusions” section) with differences in N‐linked glycans and disulfide bridge arrangements that distinguish it from other antibody isotypes. The fragment antigen‐binding region (Fab) is critical for antigen binding, neutralization and opsonization; the Fc portion is essential for initiating innate immune effector functions. Two heavy and light chains make up IgA, each folded into various globular domains including four heavy‐chain domains (VH, Cα1, Cα2 and Cα3) and two light‐chain domains (VL and CL; Figure 1).

Two IgA subclasses, IgA1 and IgA2, have been isolated from humans, gibbons, gorillas and chimpanzees and are distinguished by the length of the hinge region, numerous sequence differences in heavy‐chain constant regions and glycosylation patterns (Figure 1).^14^ However, most other nonhuman primates and mammals including mice possess one IgA subclass (IgA2 like), with the exception of orangutans which only possess IgA1.^14^ IgA1 adopts a T‐shaped formation because of an elongated hinge region including a 16‐amino acid insertion (Figure 1). IgA2 lacks this insertion and adopts a protease‐resistant closed hinge formation resulting in its characteristic Y shape (Figure 1). Currently, only one IgA1 allotype has been identified in humans and two IgA2 allotypes, namely, IgA2m(1) and IgA2m(2), which are distinguished by the presence or absence of disulfide bridges between the heavy and light chains and different glycosylation patterns,^12^ with a third possible allotype also described IgA2n.^15^ The functional differences of IgA allotypes are yet to be characterized; however, it is reasonable to predict that variation in structure of the IgA2m(1) and IgA2m(2) allotypes would influence functional characteristics similar to IgG allotypes.^16^, ^17^ Glycosylation of IgA1 differs from that of IgA2 in that three to five *O*‐linked oligosaccharides are present in the extended hinge region,^18^ affecting the hinge structure (Figure 1).^18^ Furthermore, both IgA subclasses carry N‐linked oligosaccharides making up 6–7% molecular mass of IgA1 and 8–10% of IgA2^19^ (Figure 1). Glycosylation patterns of secretory IgA (sIgA) can mediate antiviral activity.^20^ Sialic acid on the C‐terminal tail (position 459) of sIgA interacts with hemagglutinin of influenza A to disrupt cell surface attachment; however, the impact of serum IgA glycosylation for other Fc functions is poorly understood.^20^ It is interesting to speculate why evolutionarily humans have maintained both IgA1 and IgA2 subclasses, whereas most other mammals only possess an IgA2‐like subclass. We hypothesize that humans may have undergone divergent evolution from other mammals and adapted to the selection pressure on IgA1 by bacterial pathogens through evolution of IgA2. IgA2 is functionally important in mucosa, whereas IgA1 may be important for serum IgA functions (e.g. homeostasis or viral control), as reflected by differential distribution of IgA1 and IgA2. However, functional differences between IgA1 and IgA2 are yet to be fully characterized.

Heterogenous IgA molecular forms occur in humans consisting of monomeric (mIgA), dimeric (dIgA), polymeric (pIgA) and sIgA (Figure 1). These molecular forms, in addition to IgA subclasses, are differentially distributed throughout bodily compartments.^21^ In serum, IgA is primarily mIgA1 (90%) synthesized in the bone marrow and transported into the blood.^21^ Conversely, in most mucosal secretions there is a proportional increase in IgA2 because of the protease‐resistant hinge region. In addition, mucosal IgA is locally produced as dIgA in organized gut‐associated lymphoid tissues with site‐specific homing of IgA2 plasmablasts.^22^ dIgA undergoes transcytosis through epithelial cells via polymeric immunoglobulin receptor into the mucosal lumen.^21^ Throughout this process polymeric immunoglobulin receptor is cleaved, resulting in a complex consisting of dIgA and secretory component which is released as sIgA (Figure 1).^21^ Interestingly, the heterogenous forms of IgA have various roles in homeostasis and in infection.

Historically, IgA has been considered a noninflammatory antibody because of the involvement of sIgA in the downregulation of proinflammatory responses to pathogens and food antigens by preventing binding to other Fc receptors, rather than by activating anti‐inflammatory pathways such as described in a later section. The role of sIgA as a noninflammatory antibody is highlighted in sIgA‐deficient patients in whom an increased risk of autoimmunity and allergy is observed.^23^ Extensive research of mucosal secretions supports the role of sIgA in passive and potentially active immune protection of newborns within colostrum and breast milk IgA.^24^ Furthermore, adult sIgA maintains homeostasis of microbiota diversity and growth and contributes to passive immunity from invading pathogens.^11^ In comparison, the role of serum IgA (mIgA, dIgA or pIgA) is relatively understudied.

## Serum IgA and FcαRI

Recent technological developments have fostered the study of the serum IgA system in greater detail. It is clear that IgA is a poor activator of complement as it lacks a C1q‐binding site in the Fc region, although activation via the alternative and lectin pathway may be possible.^25^, ^26^ Research over the past two decades shows a dichotomous role of serum IgA in inflammation.^27^, ^28^ On the one hand, serum IgA can aid in homeostasis and anti‐inflammatory responses and, on the other hand, serum IgA can induce inflammation.^27^ Binding of IgA Fc region has been described for two IgA receptors: Fcα/μR (IgA and IgM) and Fc alpha receptor I (FcαRI).^28^ Additional IgA receptors have also been described; however, their functions are yet to be characterized.^28^

Human FcαRI (CD89) is constitutively expressed on cells of myeloid lineage including monocytes, eosinophils, some macrophages, intestinal dendritic cells, Kupffer cells and neutrophils, which are the most abundant cells in blood expressing FcαRI.^29^ FcαRI has a ligand‐binding α chain mapping to chromosome 19 with the genes for natural killer cell receptors (KIR) and leukocyte immunoglobulin‐like receptors, unlike IgG (FcγR) and IgE (FcεR) Fc receptors which map to chromosome 1.^30^ FcαRI shares closer homology with KIR and leukocyte immunoglobulin‐like receptors than other Fc receptors (e.g. FcγR).^30^ FcαRI orthologs have been identified in various other mammals including rats, chimpanzees, cattle, horses, macaques and swine; however, no known ortholog has been identified for mice.^31^, ^32^, ^33^, ^34^ Furthermore, in humans there are no reported cases of low or no FcαRI expression on myeloid cells, unlike defects reported in FcγRI which correlate with susceptibility to autoimmunity, chronic inflammation and infection,^35^ highlighting the potential importance of FcαRI in homeostasis and inflammation in humans. However, it is important to note that IgA deficiencies have been reported in humans, which have been associated with increased susceptibility to infectious diseases and autoimmunity.^36^

**Figure 2:**
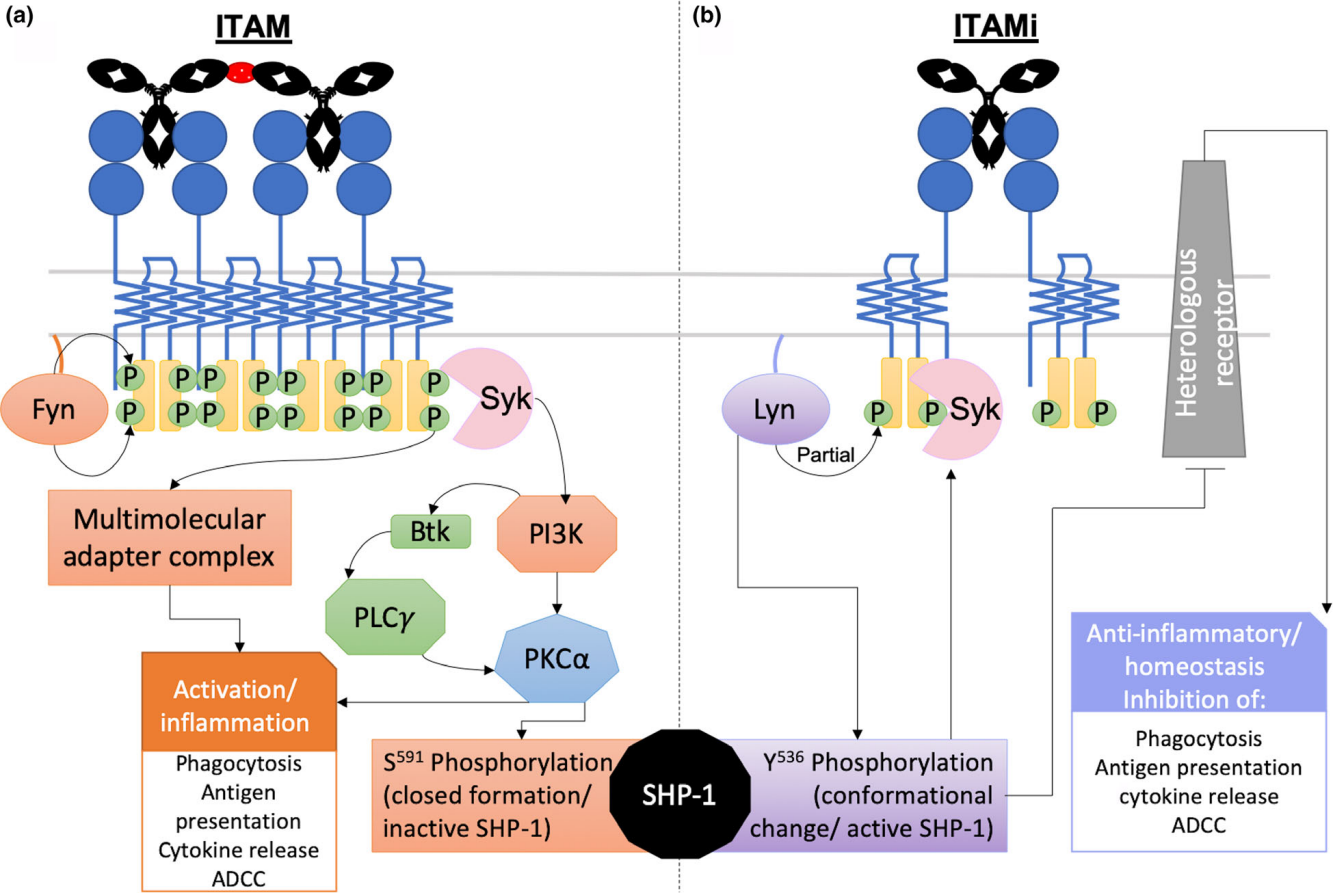
Initiation of immunoglobulin A (IgA)/Fc alpha receptor I (FcαRI) immunoreceptor tyrosine‐based activation motif (ITAM) and ITAM inhibitory (ITAMi) signal cascades and resulting Fc effector functions reviewed by Mkaddem *et al*.^42^. **(a)** IgA–antigen complex crosslinking of FcαRI initiates phosphorylation of ITAM with Fyn^43^ followed by generation of (1) multimolecular adapter complex (Cbl, SLP‐76, Grb2, CrkL, Shc, Sos, SHIP) and/or (2) recruitment of Syk and activation of phosphoinositide 3‐kinase (PI3K) which phosphorylates Btk and activates protein kinase C (PKCα). PKCα ultimately leads to activation/inflammatory effector functions and inactivation of SHP‐1 via S^591^ phosphorylation. **(b)** Uncomplexed monomeric IgA (mIgA) binding to FcαRI initiates partial phosphorylation of ITAM by Lyn, leading to ITAMi signaling. Lyn also phosphorylates SHP‐1 at Y^536^, triggering a conformational change which activates SHP‐1, leading to inhibition of heterogenous receptors, causing the cell to enter a resting state and take on homeostatic (anti‐inflammatory) functions. Phosphorylated SHP‐1 is recruited to the receptor via Syk.^43^ ADCC, antibody‐dependent cellular cytotoxicity; Fc, fragment crystallizable; PLCγ, phospholipase C‐gamma.

The FcαRI α chain has two immunoglobulins‐like extracellular domains, transmembrane region and a short cytoplasmic tail without any recognized signaling motifs.^37^ For signaling to occur FcαRI must associate with immunoreceptor tyrosine‐based activation motif (ITAM), which can be phosphorylated to initiate signal transduction. Binding of monomeric serum IgA Cα1 and Cα2 Fc domains to the membrane distal domain of FcαRI occurs in a 1:2 stoichiometry (1 IgA:2 FcαRI) as shown in Figure 2.^37^ In the presence of ITAM, binding of IgA–antigen complex to FcαRI initiates signal cascades, ultimately leading to an inflammatory response (Figure 2). However, when uncomplexed mIgA associates with FcαRI, ITAM inhibitory signal cascade is initiated, resulting in inhibition of cells and associated anti‐inflammatory/homeostatic role (Figure 2).^37^ Furthermore, two FcαRI single‐nucleotide polymorphisms have been identified in humans: Ser248/Gly248 and Asp92/Asn92. Gly248 FcαRI has been associated with increased proinflammatory potential of serum IgA^38^ and Asn92 FcαRI has been associated with increased risk of myocardial infarction.^39^ Interestingly, sIgA and dIgA bind poorly to FcαRI because of steric hindrance associated with the J chain and secretory component; however, dIgA has been reported to initiate effector functions via FcαRI against bacteria.^37^ A recurrent theme in early literature suggests that serum dIgA and pIgA enhances phagocytosis compared with mIgA, even with steric hindrance.^40^, ^41^ This may occur through FcαRI binding of dIgA at alternative binding sites, increased stability of IgA *in vitro* and a greater capacity for antigen binding because of increased valency and avidity than mIgA (Table 1).^37^

**Table 1:** Antibody properties of IgG1, IgA1, IgA2 and dIgA/pIgA in terms of effector function and viability as therapeutic monoclonal antibody.^12^, ^13^, ^16^, ^49^, ^52^, ^53^, ^54^, ^55^, ^81^, ^82^

Property	IgG1	Serum IgA		
		IgA1	IgA2	dIgA/pIgA
Half‐life	~21 days (FcRn recycling)	5.9 days	4.5 days	a
Valency/avidity	+	+	+	+++
Expression/purification	+++	+/++		+
Neutralizing/opsonization capacity	+++	+		++
Neutrophil activation	+++	+++		++
Natural killer cell‐mediated ADCC	+++	–		–
Myeloid cell‐mediated ADCC and phagocytosis	+++	++		++
Anti‐inflammatory role	+ (FcγRIIb)	+++ (FcαRI)		++ (FcαRI)
Complement activation	+++ (all pathways)	+ (potentially alternative and lectin pathways)		
Therapeutic antibody potential	+++	++		
Diseases/conditions of interest	Various infectious diseases and some cancers	Some cancers, autoimmunity/allergy and some infectious diseases		

‐, None; +, Weak; ++, Moderate; +++, Strong. ADCC, antibody‐dependent cellular cytotoxicity; FcαRI, Fc alpha receptor I; Ig, immunoglobulin.

a Contrasting literature reported.

## Antitumor role of IgA

**Figure 3:**
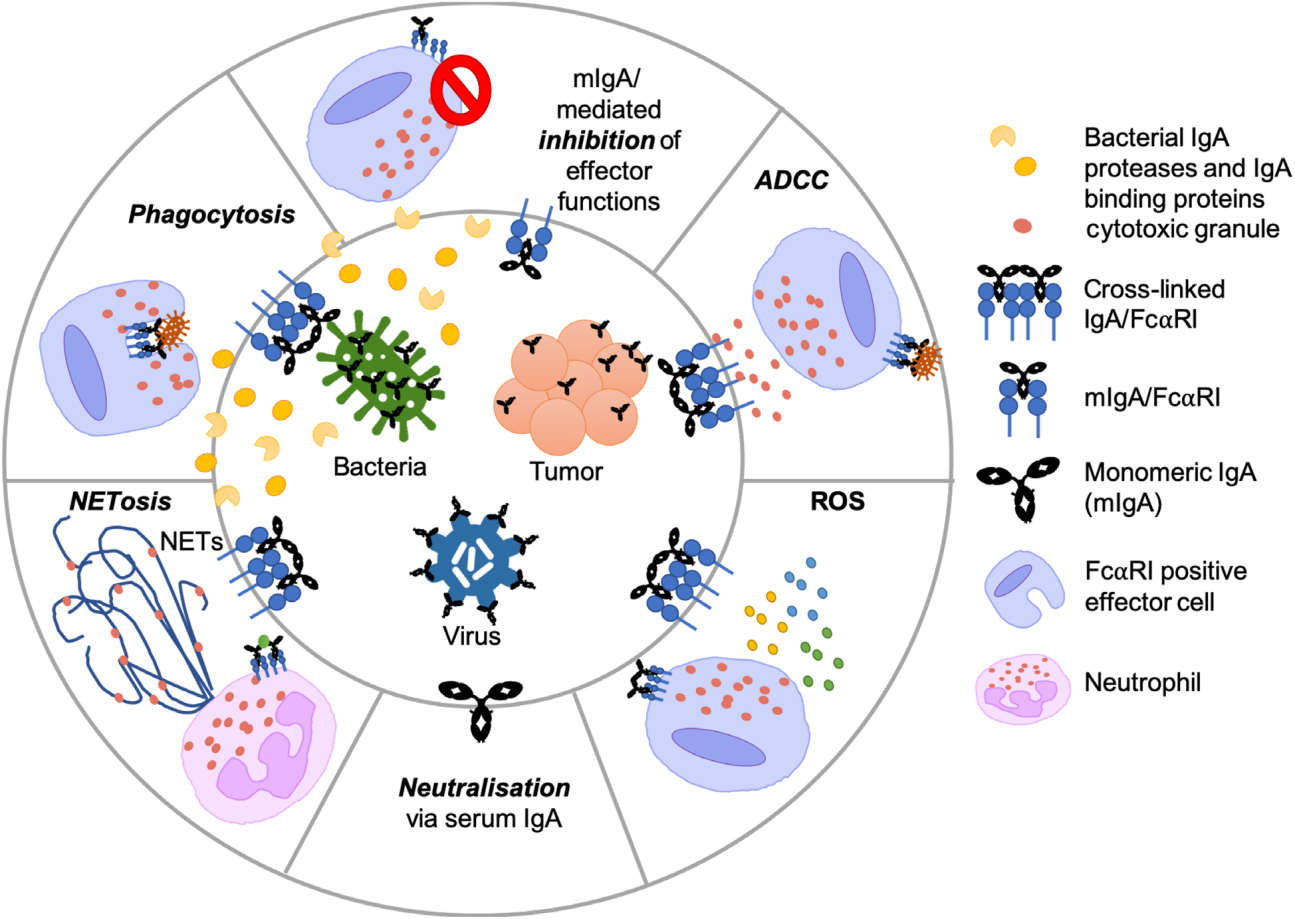
Serum immunoglobulin A (IgA) effector functions dependent and independent (neutralization) of Fc alpha receptor I (FcαRI) against bacteria, viruses and tumor cells and IgA countermeasures enabling persistence of infection. Crosslinking of FcαRI with IgA results in FcαRI‐dependent effector functions via immunoreceptor tyrosine‐based activation motif (ITAM) signaling [antibody‐dependent cellular cytotoxicity (ADCC), phagocytosis, NETosis and reactive oxygen species (ROS)]. Binding of monomeric IgA (mIgA) to FcαRI leads to ITAM inhibitory and the resulting effector cell inhibition aiding in persistence of infection/cancer. Release of anti‐IgA molecules by bacteria reduces bacterial clearance via IgA. NET, neutrophil extracellular trap.

A small number of research groups have recently focused on IgA and FcαRI engagement to treat cancer.^44^ Whereas most research has focused on IgG in mAb therapy because of potent antitumor mechanisms including complement activation and natural killer cell‐mediated ADCC, IgA appears to be potent in the recruitment and activation of neutrophils via the FcαRI to kill tumors, providing an attractive target for mAb antitumor therapy.^45^ Several neutrophil IgA‐mediated antitumor functions have been described *in vitro*, such as ADCC, phagocytosis, immune cell recruitment, release of cytotoxic molecules and induction of necrosis.^44^, ^46^ Target‐specific IgA mAbs enable formation of an immunological synapse by bringing neutrophils and target tumor cells together to enhance killing (Figure 3). Recently, IgA mAbs targeting tumor cells such as HER2 (mammary carcinoma) and CD20 (B‐cell lymphoma) have shown promising antitumor effects.^46^ Interestingly, the use of FcαRI transgenic mouse models has shown that IgA2 anti‐EGFR antibodies can induce tumor cell killing, most likely mediated by macrophages.^3^ However, more *in vivo* work is needed to dissect the contribution of FcαRI‐expressing effector cells in tumor killing. While there are several properties of IgA that make it advantageous as an antitumor mAb, IgG remains the antibody isotype of choice when it comes to mAb development as outlined in Table 1 (also reviewed elsewhere 46). Moreover, there is great debate in the field as to how effective IgA will be as an mAb therapy because high concentrations of serum IgA can be extremely detrimental as observed in the case of IgA nephropathy.^47^ Furthermore, technologies available for the expression and purification of IgA (especially dIgA/pIgA) are comparatively more complicated than IgG.^48^ However, modifications to IgA mAb can improve half‐life and stability.^49^, ^50^ Combinations of IgG and IgA mAbs can enhance tumor killing and work on “cross‐type antibodies” such as IgGA and tandem antibodies combines the best of both IgG (complement binding) and IgA (cytotoxicity/phagocytosis) antitumor effects.^51^

## Bacteria

Invasive bacterial infections can cause severe disease such as sepsis and meningitis. Early research from the 1970s through to the early 2000s highlights the role of serum IgA in the second (serum) and potentially third line (liver) of defense from bacteria that enter the blood and tissues. Killing of various bacterial species including *S. pneumoniae*, *Bordetella pertussis*, *Escherichia coli*, *Staphylococcus aureus* and *N. meningitidis* was associated with IgA‐mediated intracellular killing via phagocytosis as highlighted in various vaccine studies (Figure 3).^40^, ^41^ Johnson *et al*.^41^ observed an initial capsule‐specific serum pIgA response in both natural infection (1 month) and immunization (1–3 months). Janoff *et al*.^40^ later reported killing of *S.* *pneumoniae* via phagocytosis using human polymorphonuclear leukocytes and HL‐60s mediated through binding of capsule‐specific serum pIgA to FcαRI. Interestingly, phagocytosis of *S.* *pneumoniae* in this study also required complement as shown by inhibition of FcαRI and CD35/CD11b, where killing was reduced by 50%. Thus, killing of the *S.* *pneumoniae* in the blood involves a combination of serum pIgA/FcαRI and complement.^40^ Antibacterial phagocytosis mediated by serum IgA/FcαRI has been observed against *B.* *pertussis* in FcαRI transgenic mice using IgA‐coated *B.* *pertussis* with human polymorphonuclear leukocyte, leading to enhanced bacterial clearance in the lungs.^6^ The phagocytic role of serum IgA in other bacterial species is more controversial, like that of *Neisseria* spp., the causative agents of gonorrhea (*N.* *gonorrhoeae*) and meningitis (*N.* *meningitidis*).^7^ Some studies have reported IgA‐opsonized bacteria being phagocytosed, whereas others fail to observe such a phenomenon.^7^, ^56^, ^57^ Under “normal” conditions (not vaccine studies) serum IgAs often fail to induce phagocytosis of *Neisseria* spp. and we now understand that this is because of secretion of anti‐IgA molecules discussed below (see the “Anti‐IgA Mechanisms” section).^56^ Furthermore, the role of IgA in the third line of defense was demonstrated in an *in vivo* study using Kupffer cells of the liver which naturally express FcαRI. van Egmond *et al*.^58^ observed efficient removal of serum IgA‐opsonized *E.* *coli* from portal circulation mediated by interaction between serum IgA (mIgA, dIgA and pIgA) and FcαRI. It is evident from existing research that serum IgA and FcαRI have the potential to initiate phagocytosis of IgA‐opsonized bacteria.

Serum IgA can induce additional effector functions such as ADCC and powerful neutrophil effector functions, although limited literature describes such processes in bacterial infection (Figure 3).^37^ ADCC has been observed to occur using vaccine‐induced sIgA and serum IgA against various bacterial species including *Salmonella enterica* serotype Typhi.^59^ Interestingly, other structures such as neutrophil extracellular traps (NETs) may also be key to IgA/FcαRI role in bacterial infection. NETs are web‐like structures extruded by neutrophils trapping and killing pathogens.^60^ NET formation can occur in two forms: rapid formation within minutes independent of reactive oxygen species or slow formation over several hours dependent on generation of reactive oxygen species, resulting in cell membrane rupture and cell death, commonly referred to as NETosis.^61^ Recently, Aleyd *et al*.^60^ observed that *S.* *aureus* opsonized with IgA resulted in NETosis via the FcαRI, compared with non‐IgA‐opsonized bacteria which did not. The study of serum IgA in vaccine settings has highlighted the potential of IgA Fc effector function in bacterial clearance. However, in natural infection, as briefly mentioned previously, regarding *Neisseria* spp., bacteria can overcome the antibacterial Fc effector functions of serum IgA.^56^, ^62^

### Anti‐IgA mechanisms

Evolution of anti‐IgA bacterial mechanisms is a unique feature of many pathogenic bacteria highlighting the importance of IgA in the control and clearance of invasive bacterial diseases including *N.* *meningitidis*, *Haemophilus influenzae* and group A and B streptococci. Two such mechanisms include IgA proteases and IgA‐binding proteins (Figure 3). Interestingly, such anti‐IgA mechanisms are yet to be reported for viruses, although some viruses have evolved to secrete FcγR‐blocking proteins.^63^ This suggests that IgG‐mediated Fc functions may evolutionarily be more efficient at viral control than IgA‐mediated Fc mechanisms. Furthermore, evolution of alternative mechanisms, such as B‐cell dysfunction in HIV, ultimately disrupts antibody maturation as a whole, including the function of IgA.^64^

#### Bacterial mechanisms

##### IgA proteases

IgA1 proteases are secreted by many bacterial pathogens including *N.* *meningitidis*, *H. influenzae* and *S.* *pneumoniae* to aid invasion into tissues and potentially the blood leading to septicemia and bacterial meningitis. These enzymes cleave the exposed hinge region of IgA1 at various different sites including specific Pro–Ser or Pro–Thr peptide bond.^62^ Furthermore, cleaved IgA1 may compete for functional antibodies via binding of the fragment antigen‐binding region to antigen preventing binding of intact antibodies.^62^ These proteins have arisen through convergent evolution and are associated with virulence.^65^ Closely related strains of these bacteria lacking IgA1 proteases are nonvirulent.^65^ Interestingly, some bacteria including *Pseudomonas aeruginosa* secrete broad‐spectrum proteases that can cleave IgA1 and IgA2. Although IgA2 possesses a closed and more protected hinge region, bacteria such as *Clostridium ramosum* and *Pasteurella multocida* secrete proteases that cleave IgA2m(1) and IgA2m(2), respectively.^66^, ^67^

##### IgA‐binding proteins

Another evasion mechanism present in bacteria is IgA‐binding proteins expressed by many strains of group A and B streptococci. *Streptococcus* group A possess Arp4 and Sir22 (M peptide family) IgA‐binding proteins are associated with virulence and group B *Streptococcus* has an unrelated β protein.^68^ These proteins interact with the Fc interdomain region between the Cα2 and Cα3 domains, competing for FcαRI binding, and inhibit IgA Fc functions in natural infections.^68^ An IgA‐binding protein has also been identified in pathogenic *E.* *coli* (EsiB) which impairs neutrophil activation via IgA.^57^ Vaccination and mAb therapy aiming to increase serum IgA levels may overwhelm bacterial evasion mechanisms and thus induce effective clearance of bacteria via IgA/FcαRI activation. However, prolonged elevation of IgA levels may be detrimental in the long term^42^ (see the “The Future of IgA in Infectious Disease mAb Therapy” section).

## Viruses

Although research into the role of serum IgA in viral infections is less comprehensive than bacterial infection, the potential for serum IgA to mediate protection is highlighted in rotavirus and HIV infections. In various rotavirus vaccine trials, serum IgA has been established as a correlate of protection for vaccine efficacy in a systemic review of antirotavirus serum IgA titers of Rotarix (RV1) and RotaTeq (RV5) vaccines.^69^ Patel *et al*.^69^ proposed that serum IgA titers >90 postvaccination showed a significant increase in efficacy of the vaccines. However, a study with children from the United States showed that greater IgA titers (>200) correlated with protection from natural infection.^70^ As for the mechanism of protection, it has been hypothesized that the serum and/or sIgA may neutralize rotavirus.^71^ However, work using IgA mAbs directed against the intermediate capsid protein VP6 of rotavirus in mice did not neutralize the virus, but inhibition of viral transcription in epithelial cells was observed.^4^ The role of serum IgA Fc functions in rotavirus protection and clearance is yet to be reported.

### HIV

The protective potential of serum IgA has been suggested in elite controllers (individuals that spontaneously control HIV‐1 viremia) in whom higher titers of HIV‐1‐specific serum IgA have been observed compared with HIV‐1 progressors.^72^ *In vitro* studies have demonstrated that monoclonal IgA has the capacity to activate antibody functions against HIV‐1 antigens (ADCC and phagocytosis).^10^, ^73^ Furthermore, mucosal IgA (sIgA) may prevent HIV‐1 infection via immune exclusion/opsonization as observed in highly exposed seronegative individuals and various nonhuman primate vaccine trials (Figure 3).^74^

However, the role of serum IgA in HIV‐1 infection is controversial. This was highlighted by the protective RV144 human HIV‐1 vaccine trial (31.2%) in which HIV‐1‐specific IgG was associated with ADCC and protection from HIV‐1 infection in vaccinated individuals.^75^ However, RV144‐induced serum IgA was associated with reduced ADCC and vaccine efficacy, as a result of IgA epitope competition with protective HIV‐1‐specific IgG for the same binding site on HIV envelope proteins.^52^ Interestingly, low titers of HIV‐1‐specific antibodies are produced especially during chronic infection.^74^ This suggests that the probability of HIV‐specific IgA complexed with HIV‐1 binding to FcαRI, initiating ITAM signaling and associated effector cell functions, may be very low. Furthermore, uncomplexed serum mIgA may initiate ITAM inhibitory signaling via FcαRI, dampening inflammatory cellular effector functions, thus polarizing the immune response to an anti‐inflammatory response and hindering viral clearance (Figure 3).

### The future of IgA in infectious disease mAb therapy

In the era of mAb therapy, IgA may provide a viable alternative to IgG mAb for various bacterial and viral diseases including *M. tuberculosis*, the causative agent of tuberculosis. In 2011, FcαRI transgenic mice showed protection against tuberculosis after being given a novel human IgA (monomeric IgA1) mAb as part of passive immunotherapy.^5^ Balu *et al*.^5^ hypothesized that binding of mIgA complexed with *M.* *tuberculosis* to FcαRI‐positive alveolar macrophages and/or neutrophils activated antibacterial activity of the infected cells. As knowledge of chimeric IgA mAb design for cancer therapy increases, researchers can begin tailoring of mAbs for bacterial clearance such as increasing resistance to bacterial proteases and IgA‐binding proteins and enhancing activation of potent IgA Fc effector functions.

### Limitations of IgA in infectious diseases and mAb therapy

Although mice models have been extensively used in the study of serum IgA and mAb therapy, there are several substantial differences in the IgA systems between humans and mice, as summarized in Table 2. Although recombinant FcαRI mice models have been created, translation of serum IgA research in transgenic mice infection to human infections should be interpreted carefully.

**Table 2:** Characteristics of human and mouse serum IgA systems.

	Human	Mouse
Isotypes	Two (IgA1 and IgA2)^21^	One^21^
Major molecular form of serum IgA	Monomeric (IgAI)^21^	Dimeric^21^
Presence of Fcα/μR (CD351)	Yes^28^	Yes^76^
Presence of FcαRI (CD89)	Yes^28^	No^77^
Ability to bind bacterial IgA‐binding proteins	Yes^78^	No^78^
Human IgA half‐life	4–6 days^79^	10–14 h^80^

IgA, immunoglobulin A; FcαRI, Fc alpha receptor I.

IgA autoantibodies have been reported as the mediator for several diseases including IgA nephropathy (elevated IgA levels), rheumatoid arthritis, coeliac disease and various IgA‐associated skin diseases reviewed by Heineke and van Egmond.^44^In many of these cases, elevated IgA levels coincide with increased IgA autoantibodies, resulting in high levels of inflammation including excessive activation of neutrophils. This suggests that prolonged elevation of serum IgA, especially IgA targeting self‐antigens, can have dire consequences. Therefore, extreme care should be taken when developing IgA mAbs and a personalized medicine approach may need to be considered based on basal serum IgA levels and IgA autoantibody levels to maintain a healthy balance between inflammation and anti‐inflammatory mechanisms.

## Future Directions and Conclusions

Creating a balance between inflammatory response to clear infection while not inducing an over inflammatory environment is crucial to effective serum IgA response to pathogenic infections. However, further research into FcαRI signaling pathways (ITAM and ITAM inhibitory) is critical to understand this balance. Uncovering how these pathways moderate inflammation, downregulate the overall activation of effector cells and discovering if this is associated with persistence of infection, will give researchers insight into the importance of serum IgA in infection. Furthermore, the role of IgA/FcαRI in infectious disease appears to vary between pathogens (bacterial or viral) and between species (e.g. HIV and rotavirus). Thus, IgA/FcαRI level of activation and/or inhibition should be characterized independently for each pathogen to confirm the respective roles of IgA function for specific infections. Renewed research will provide valuable insights regarding the therapeutic potential of serum IgA.

## Conflict of Interest

None declared.
